# Supplementary material for: Predictive Analyses of Prognostic-Related Immune Genes and Immune Infiltrates for Glioblastoma
Source: Diagnostics (Basel). 2020 Mar 24;10(3):177. doi: 10.3390/diagnostics10030177 (PMC7151008; doi:10.3390/diagnostics10030177)
Supplement: Supplementary file 1 [file diagnostics-10-00177-s001.zip › diagnostics-703991-supplementary/Figure S10.docx]

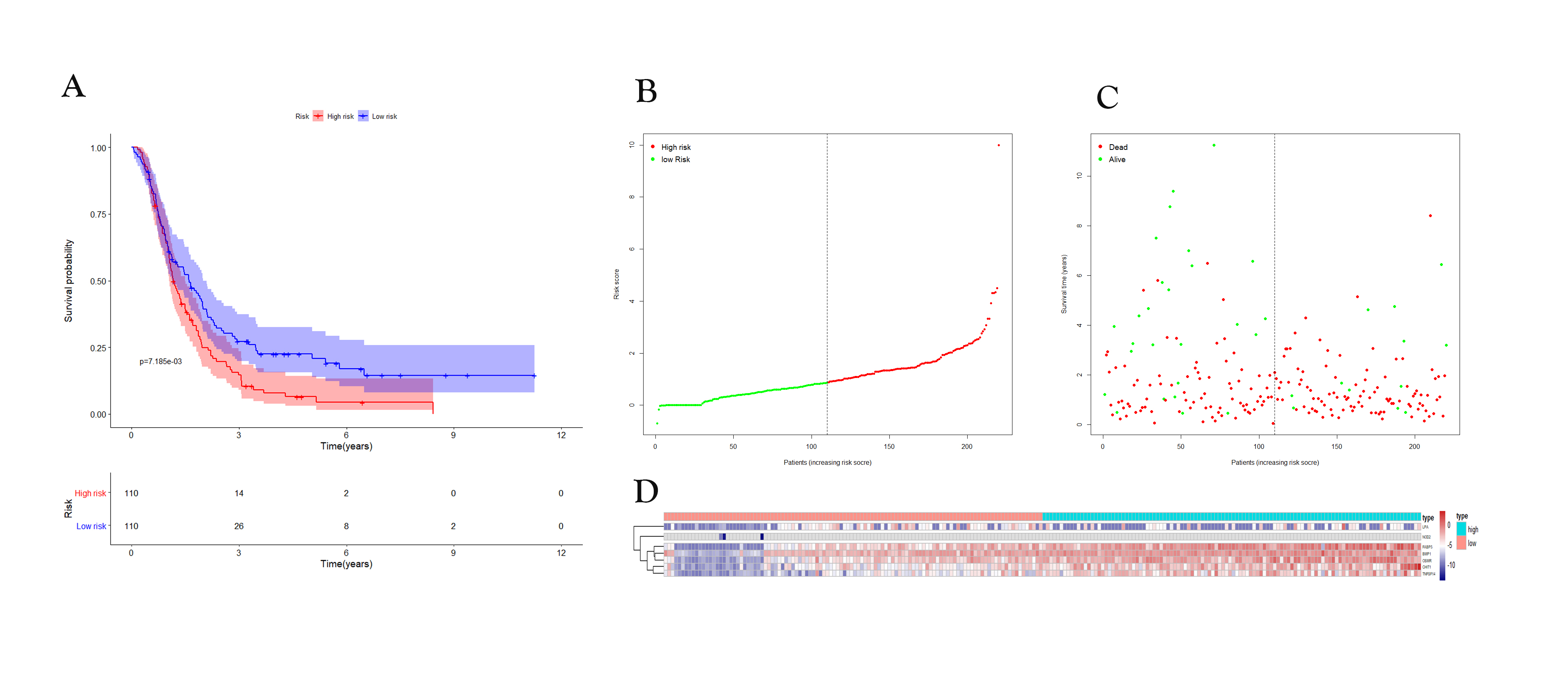


**Figure S10**. **A** The K-M survival curve shows the difference between the low-risk group and high-risk group patients. **B** The survival was prolonged in low-risk patients compared to the high-risk group. **C** The survival was prolonged in low-risk patients compared to the high-risk group. **D** The changes in immune gene expression are shown based on the risk score.
