# Supplementary figures and images for: Predictive Analyses of Prognostic-Related Immune Genes and Immune Infiltrates for Glioblastoma
Source: Diagnostics (Basel). 2020 Mar 24;10(3):177. doi: 10.3390/diagnostics10030177 (PMC7151008; doi:10.3390/diagnostics10030177)

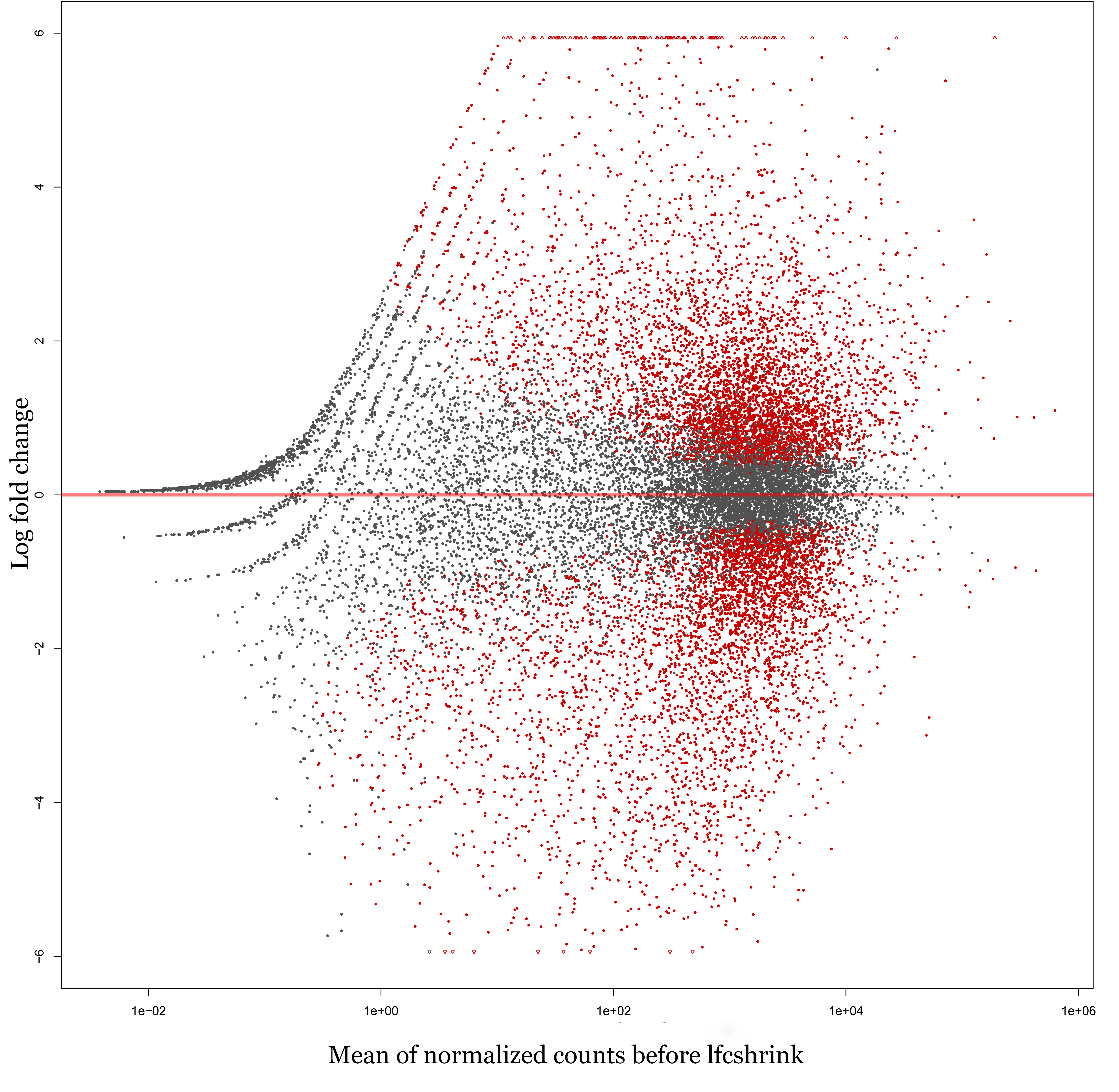

Supplement: Supplementary file 1 [file diagnostics-10-00177-s001.zip › diagnostics-703991-supplementary/Figure S1.tif]

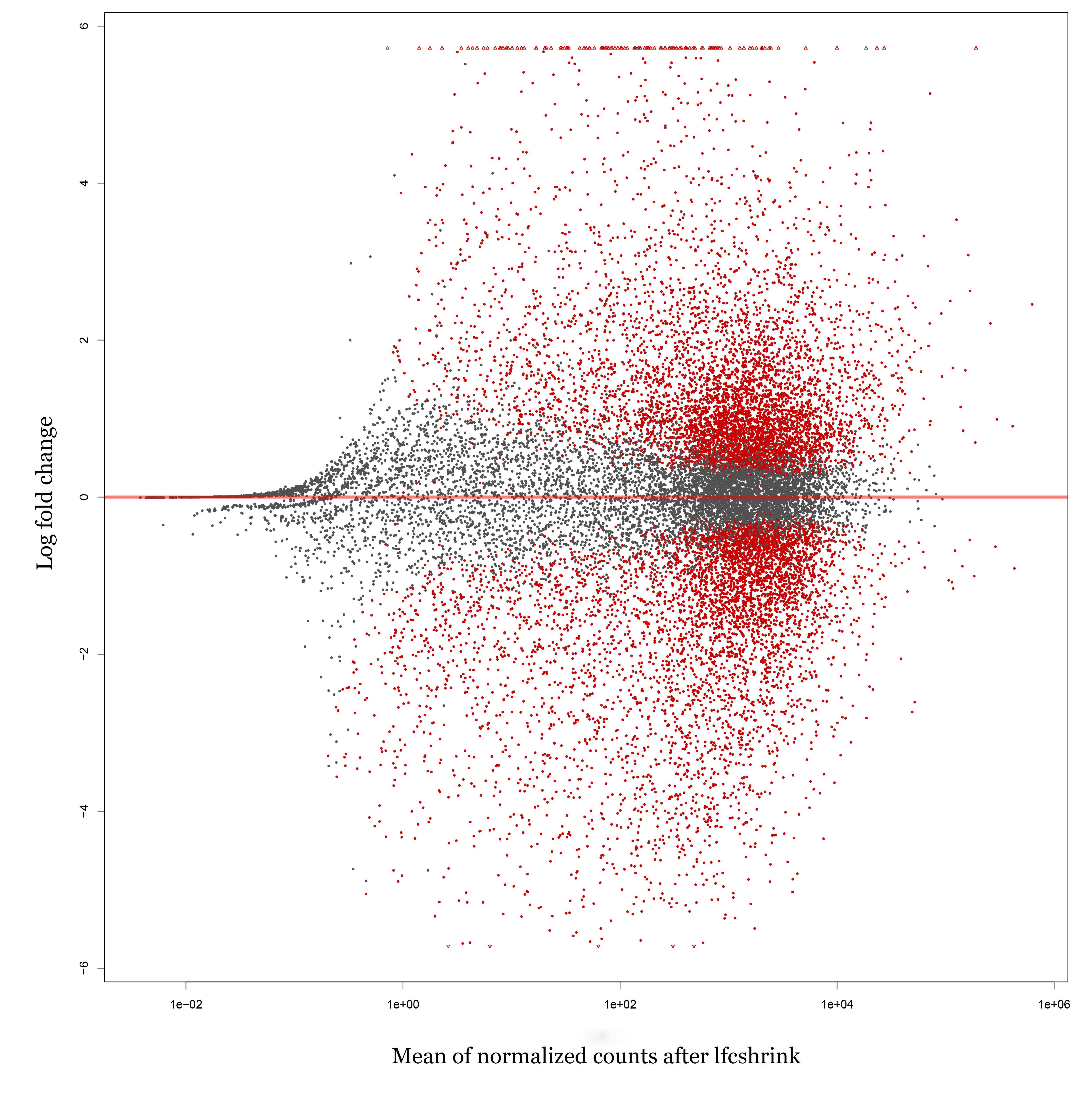

Supplement: Supplementary file 1 [file diagnostics-10-00177-s001.zip › diagnostics-703991-supplementary/Figure S2.tif]

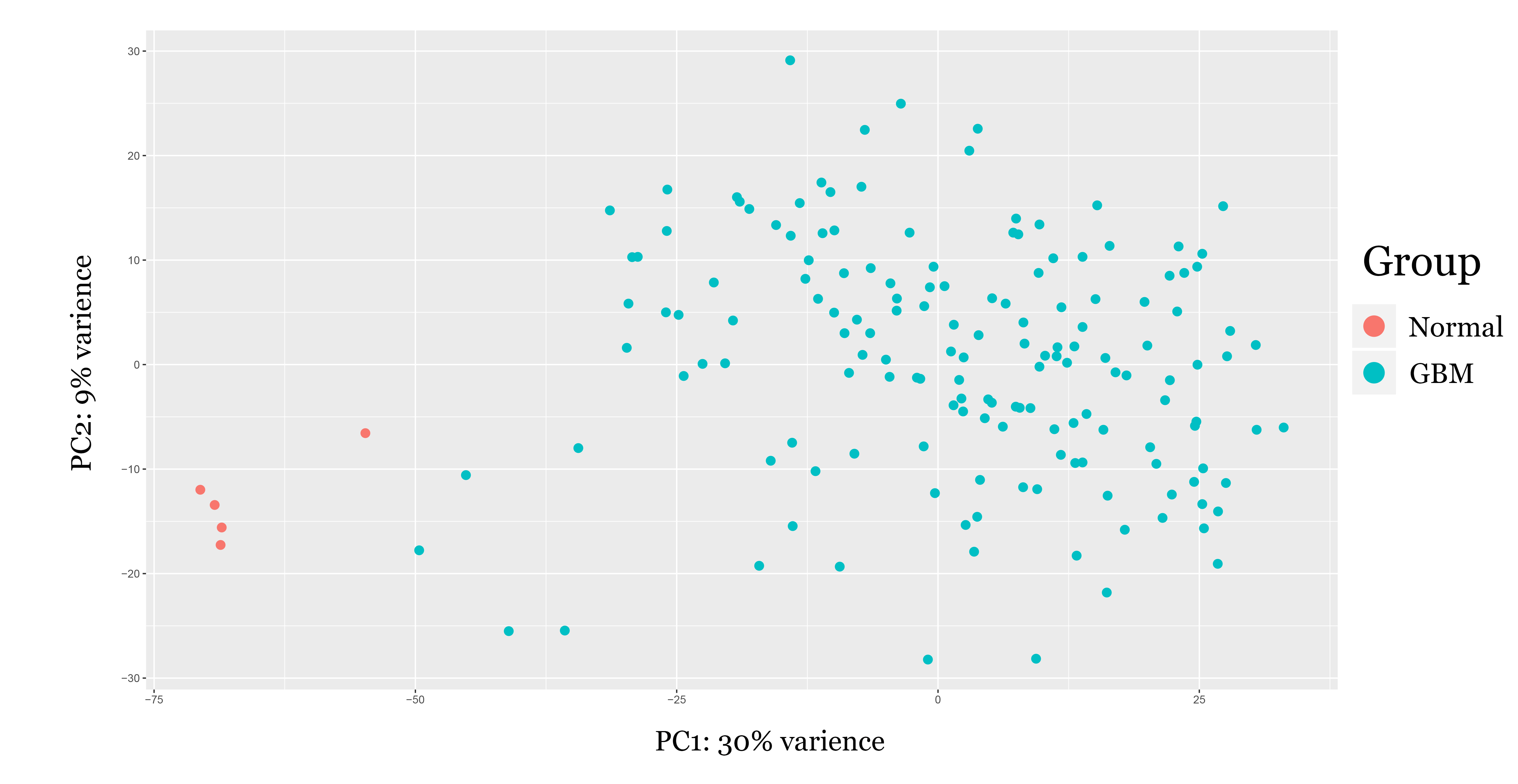

Supplement: Supplementary file 1 [file diagnostics-10-00177-s001.zip › diagnostics-703991-supplementary/Figure S3.tif]

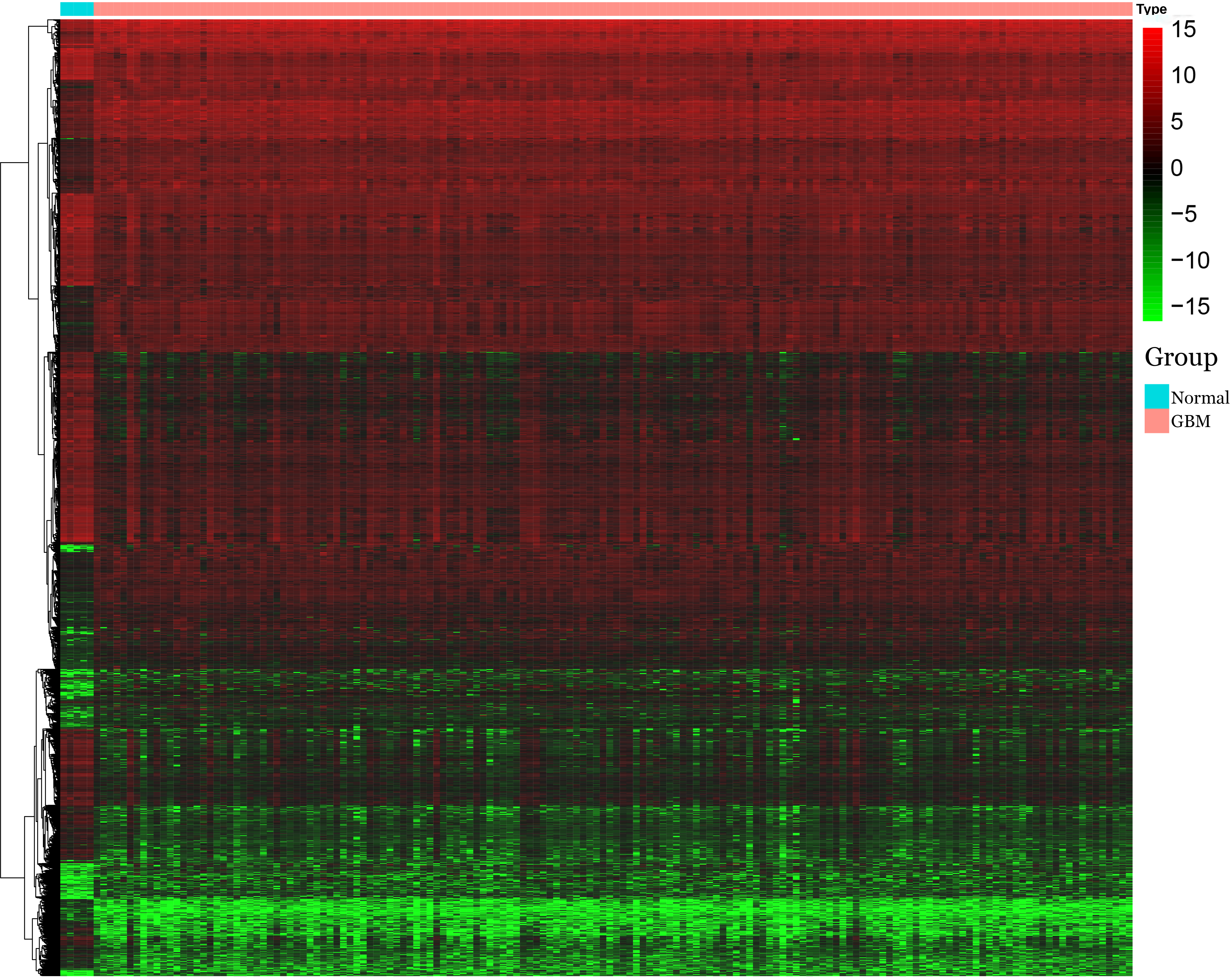

Supplement: Supplementary file 1 [file diagnostics-10-00177-s001.zip › diagnostics-703991-supplementary/Figure S4.tif]

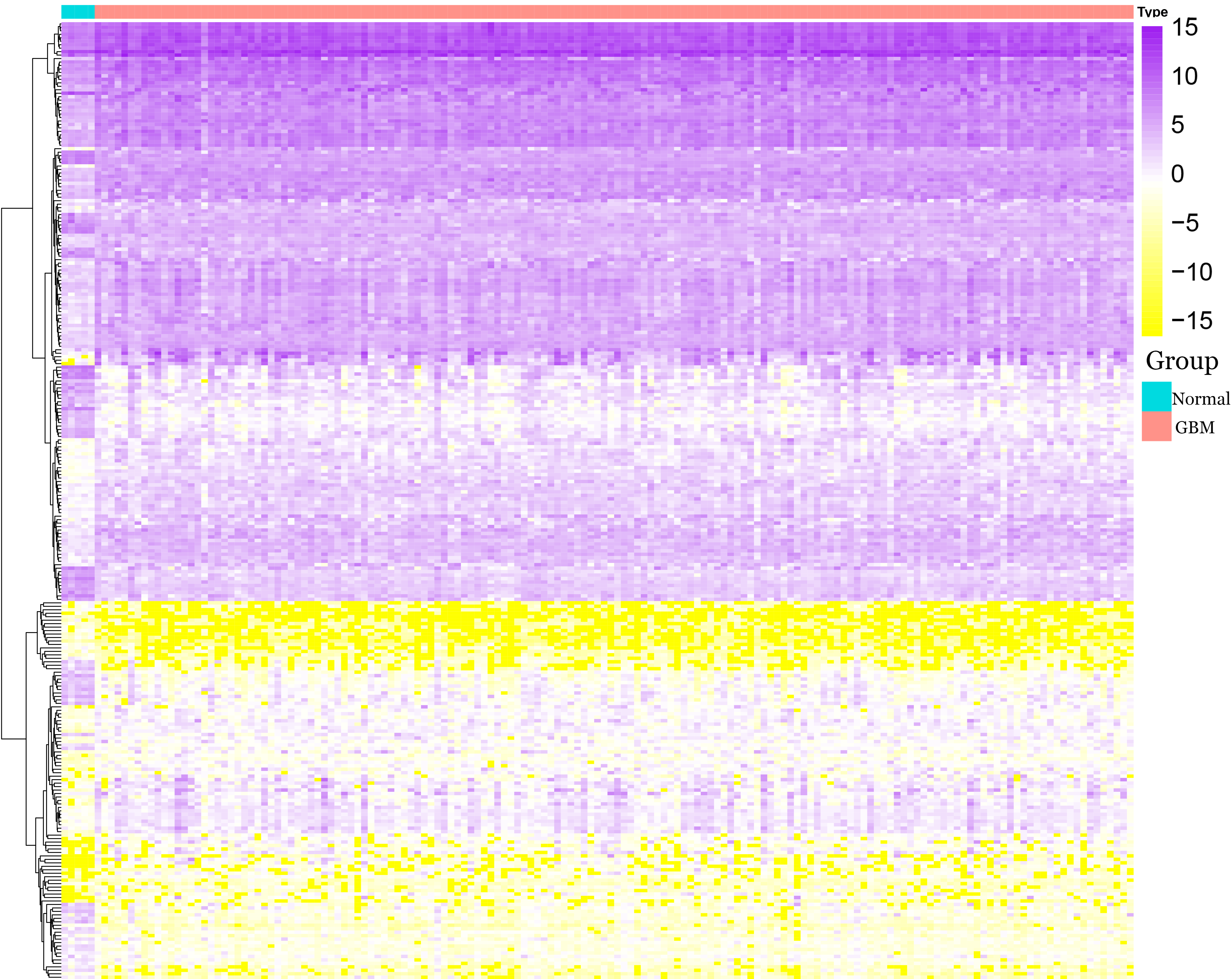

Supplement: Supplementary file 1 [file diagnostics-10-00177-s001.zip › diagnostics-703991-supplementary/Figure S5.tif]

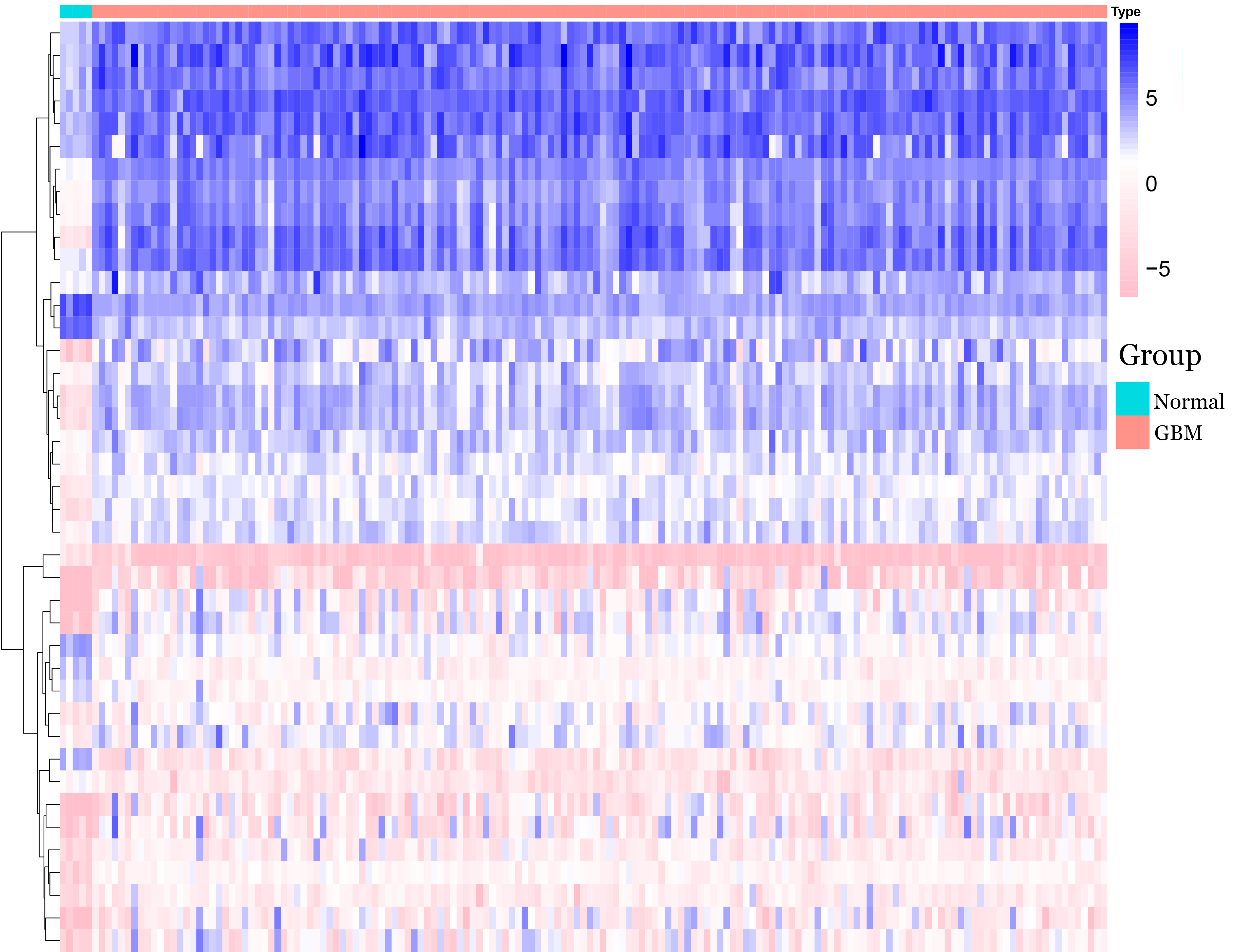

Supplement: Supplementary file 1 [file diagnostics-10-00177-s001.zip › diagnostics-703991-supplementary/Figure S6.tif]

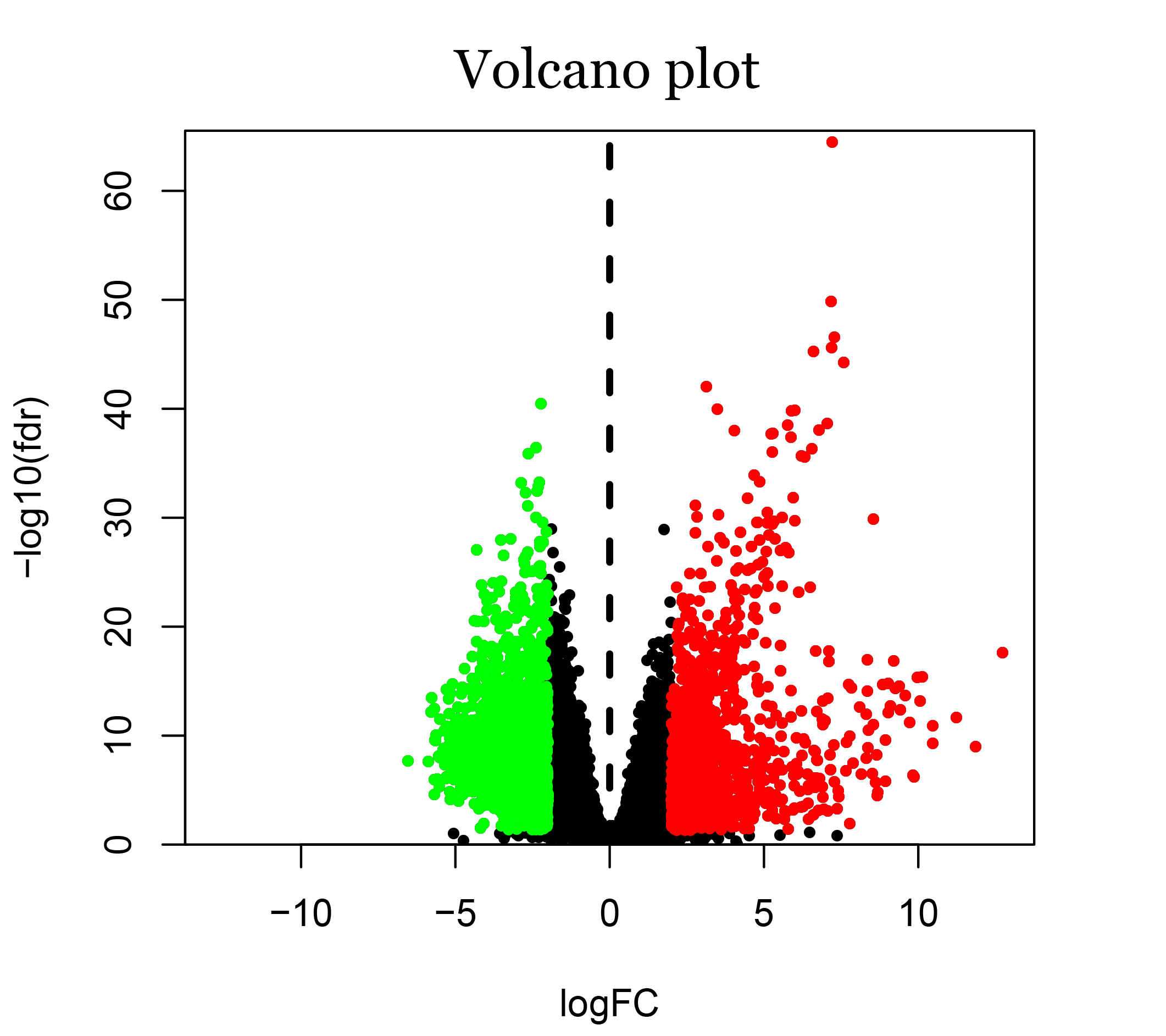

Supplement: Supplementary file 1 [file diagnostics-10-00177-s001.zip › diagnostics-703991-supplementary/Figure S7.tif]

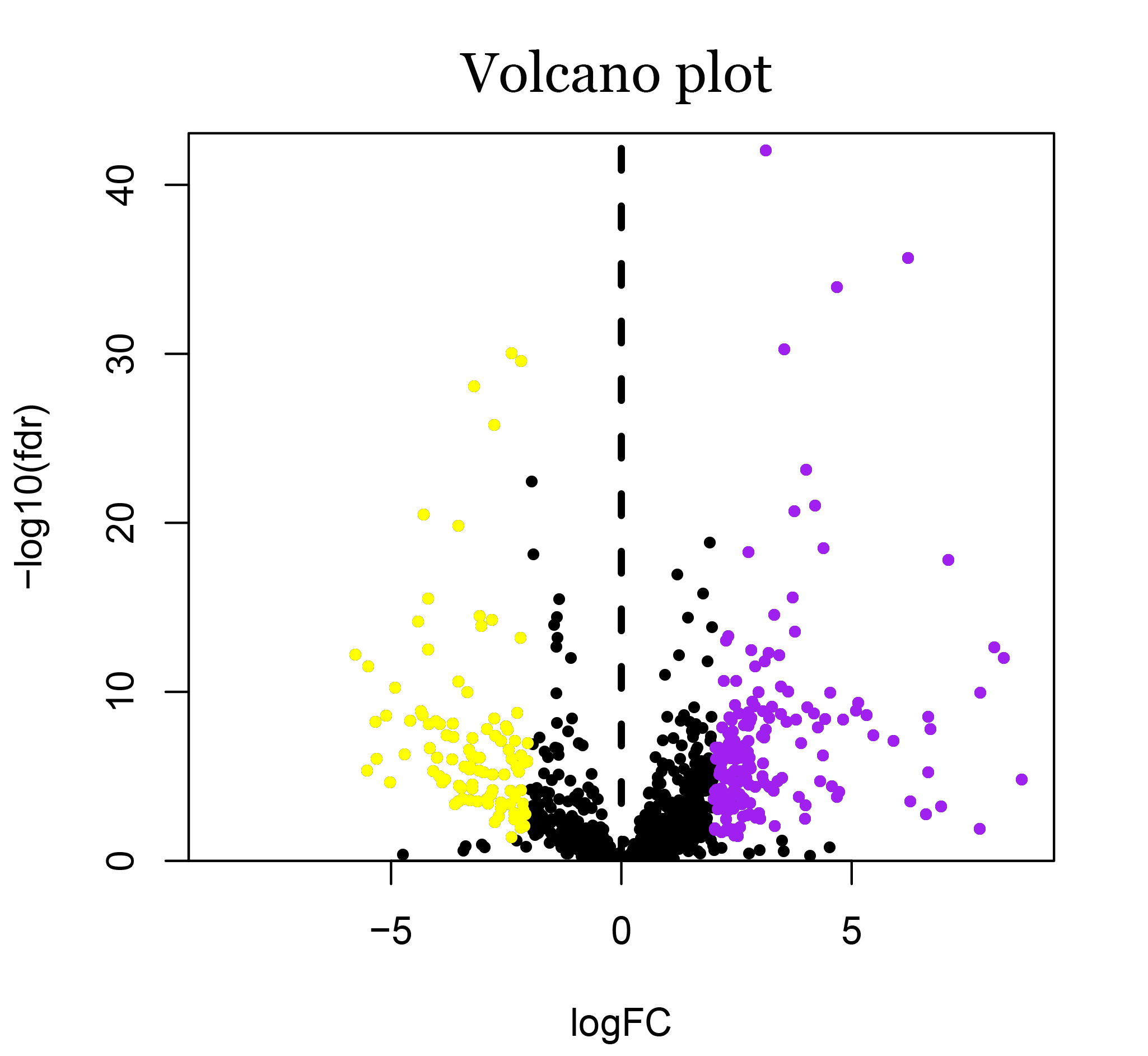

Supplement: Supplementary file 1 [file diagnostics-10-00177-s001.zip › diagnostics-703991-supplementary/Figure S8.tif]

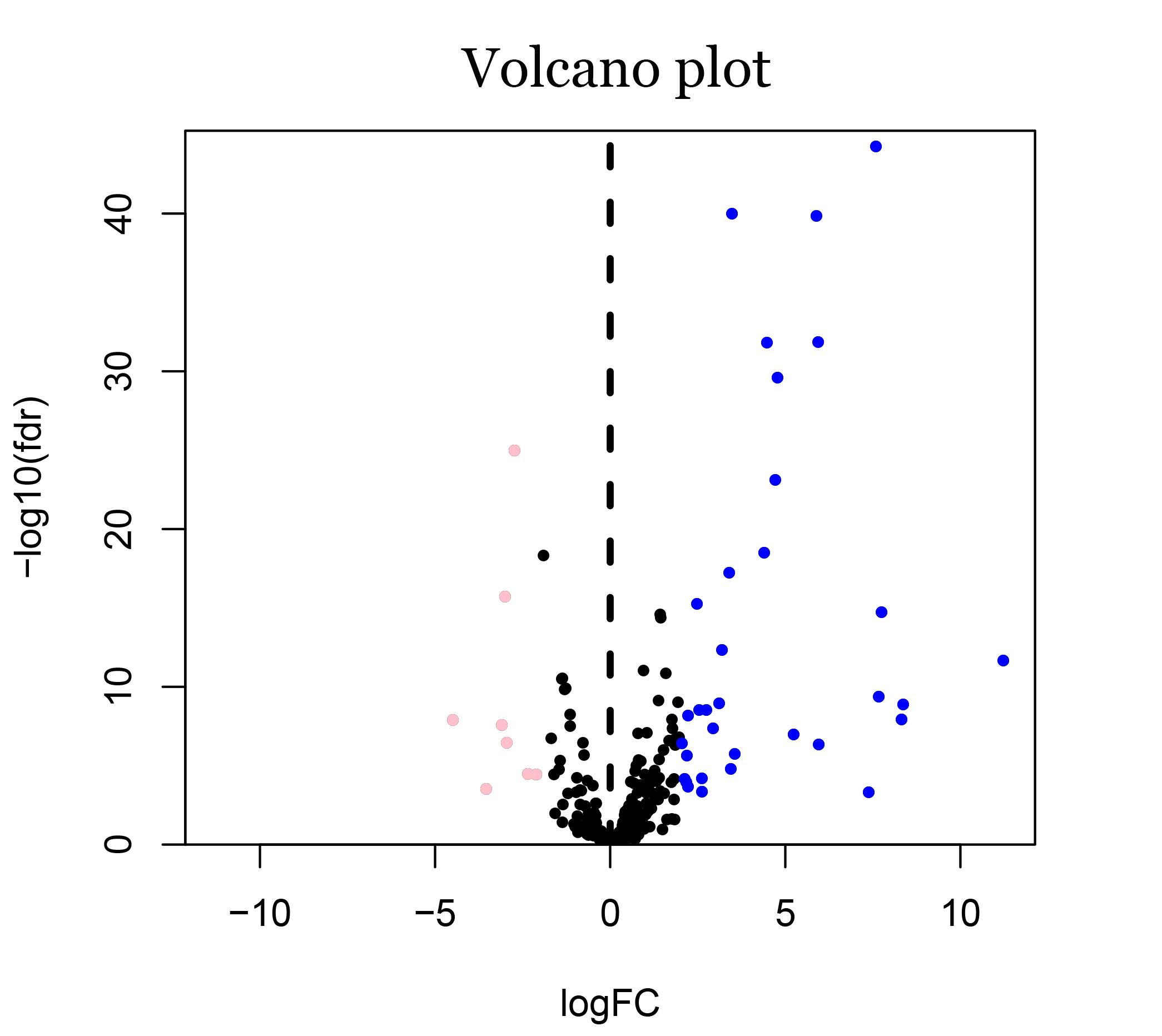

Supplement: Supplementary file 1 [file diagnostics-10-00177-s001.zip › diagnostics-703991-supplementary/Figure S9.tif]
